# The silicon efflux transporter BEC1 is essential for bloom formation and stress tolerance in cucumber

**DOI:** 10.1111/jipb.13917

**Published:** 2025-05-06

**Authors:** Changxuan Xia, Aijun Mao, Shanshan Yin, Huitong Teng, Caijiao Jin, Jian Zhang, Ying Li, Rui Dong, Tao Wu, Changlong Wen

**Affiliations:** ^1^ Beijing Vegetable Research Center (BVRC) Beijing Academy of Agriculture and Forestry Sciences Beijing 100097 China; ^2^ State Key Laboratory of Vegetable Biobreeding National Engineering Research Center for Vegetables Beijing 100097 China; ^3^ Key Laboratory of Biology and Genetic Improvement of Horticultural Crops, Key Laboratory of Superior Quality Vegetable Germplasm Innovation Ministry of Agriculture and Rural Affairs Beijing 100097 China; ^4^ Beijing Key Laboratory of Vegetable Germplasms Improvement Beijing 100097 China; ^5^ College of Horticulture Hunan Agricultural University Changsha 410128 China

**Keywords:** bloomless cucumber, graft, Si transporter, silicon, stress

## Abstract

Silicon (Si) plays a crucial role in plant growth, development, and stress tolerance. However, in some consumable plant products, such as fruits, Si deposition leads to the formation of a white powdery layer known as bloom, which diminishes glossiness and consumer appeal. Despite its significance, the genetic basis of bloom formation remains largely unexplored. Here, we identified a unique cucumber backbone parent line exhibiting bloomless fruit, which was designated *blooml ess cucumber 1* (*bec1*). Map‐based cloning of the *bec1* locus revealed that *BEC1*, harboring a natural C‐to‐T variation at the 754th base of its coding region, is a strong candidate gene for the bloomless trait. Functional validation through gene‐editing mutants and *BEC1::BEC1‐GFP* transgenic lines confirmed that *BEC1*, encoding a Si efflux transporter, is responsible for bloom formation. Mutation of *BEC1* impaired Si uptake, thereby preventing the deposition of Si on the surface of glandular trichomes and resulting in bloomless fruits. Additionally, Si deficiency in *BEC1* mutants compromised resistance to *Corynespora cassiicola* and chilling stress. Interestingly, grafting *bec1* scions onto bloom rootstocks restored the Si accumulation and stress resistance, while maintaining bloomless phenotype. Overall, our findings elucidate the role of *BEC1* in bloom formation and provide a valuable genetic target for breeding bloomless cucumber with enhanced stress resilience.

## Graphical abstract

Mutation of the silicon efflux transporter gene *BEC1* in cucumber impaired silicon uptake, resulting in fruits lacking bloom, the white powdery layer on fruits, and compromising stress resistance. Grafting *bec1* scions onto bloom‐competent rootstocks restored the silicon accumulation and stress resistance, while maintaining the bloomless phenotype.

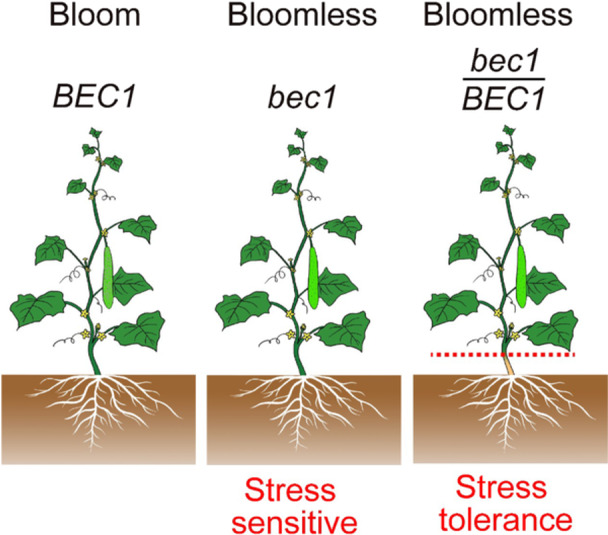

## INTRODUCTION

Silicon (Si) is the most abundant mineral element in the soil and can be deposited in different parts of the plant, like roots, leaves, and fruits (Mandlik et al., 2020). The deposition of Si affects growth and development, as well as mitigates the damage caused by biotic and abiotic stress in plants (Ma, 2004; Coskun et al., 2019; Mitani‐Ueno et al., 2023). In particular, Si deposits on consumable products such as fruits of plants polymerize into SiO_2_ and result in a layer of white powder known as bloom (Samuels et al., 1991; Motomura et al., 2002; de Melo et al., 2010). This phenomenon is observed in various plants, such as grapes, blueberries, plums, loquat, and Cucurbitaceae (Samuels et al., 1991; Takeda et al., 2013; Abe, 2019; Mandlik et al., 2020). However, this process has been paid lesser attention, even though the bloom predominantly affects the glossiness of products and their consumption characterization (Takeda et al., 2013; Abe, 2019). Like cucumbers, the Si deposition in the glandular trichomes on the fruit's surface leads to bloom fruit, making them unable to satisfy the consumer preference for bloomless fruit for its glossiness and attractive appearance (Samuels et al., 1993; Mitani et al., 2011; Tripathi et al., 2017). Therefore, molecular genetic tools have been increasingly sought to create bloomless cucumbers.

It is well known that Si is taken up as silicic acid Si(OH)_4_, mediated by Si influx and efflux transporters, respectively (Mitani‐Ueno et al., 2023). The Si influx transporter (OsLsi1 in rice) first imports Si from the soil into the symplast of roots (Ma et al., 2006), then the Si efflux transporter (OsLsi2 in rice) exports Si across the Casparian strip to the stele (Ma et al., 2007). After uptake, the Si is rapidly released to the xylem by both Lsi2 and Lsi3 (a homolog of Lsi2); then another Si influx transporter (OsLsi6 in rice) unloads Si from the xylem and delivers it to the different aerial parts of the plant (Yamaji et al., 2008; Huang et al., 2022). However, the Si transporters that take up Si from the soil and finally transport it to the surface of glandular trichomes to form bloom in cucumber have not yet been identified (Mandlik et al., 2020; Hao et al., 2024). Therefore, it has become essential to characterize the Si transporter in cucumber for breeding bloomless fruit.

The Si influx and efflux transporter genes are reported to affect Si accumulation and resistance to plant stress. In detail, the mutation of *OsLsi1* results in a negligible accumulation of Si in the shoot, which is susceptible to blast fungus (Ma et al., 2006; Ueno et al., 2007). Overexpressing *OsLsi1* in rice improved the cold tolerance by accumulating more Si in the leaf (Azeem et al., 2016). The *OsLsi2* mutant was found to have lower Si accumulation and produced a reduced grain yield under field conditions, probably due to decreased resistance to stress (Ma et al., 2007; Coskun et al., 2021). Knockout of *OsLsi6* in rice resulted in a lesser distribution of Si into the panicles, but more into the flag leaf, which increased the resistance to various stresses (Yamaji et al., 2008). Knockout of another efflux transporter (*SIET4*) induces abnormal Si deposition in mesophyll cells and the induction of hundreds of genes related to various stress responses (Mitani‐Ueno et al., 2023). In addition, a loss of function of a Si influx transporter (CmLsi1) in pumpkin rootstock (bloomless pumpkin rootstock) led to cucumber scion significantly reduced Si accumulation in leaves (Seki and Hotta, 1997; Mitani et al., 2011). Notably, Si accumulation in cucumber was essential for resistance to multiple stresses, such as downy mildew, *Pythium ultimum*, drought, salinity, and cold (Chérif et al., 1992; Fawe et al., 1998; Ma et al., 2004, 2023; Zhu et al., 2020). Thus, it became imperative to explore the manipulation of Si transport for resistant plants with bloomless fruits.

Here, we identified a backbone parent line characterized by bloomless and glossy fruit, designated as *bloomless cucumber 1* (*bec1*). The *bec1* locus was map‐based cloned using an F_2_ population. Phenotypic analysis of transgenic cucumber revealed that the major gene *BEC1* determined the formation of bloom and encodes a Si efflux transporter. The mutation of *BEC1* inhibited the uptake and transport of Si resulting in bloomless fruits, which impaired resistance to *Corynespora cassiicola* and chilling stress. Interestingly, grafting *bec1* scions onto rootstocks restored the Si and resistance deficiency, and still produced bloomless fruits. These findings provide valuable genetic resources and gene targets for breeding bloomless with enhanced stress tolerance.

## RESULTS

### A cucumber backbone parent line *bec1* exhibits bloomless and glossy fruit

**Figure 1:**
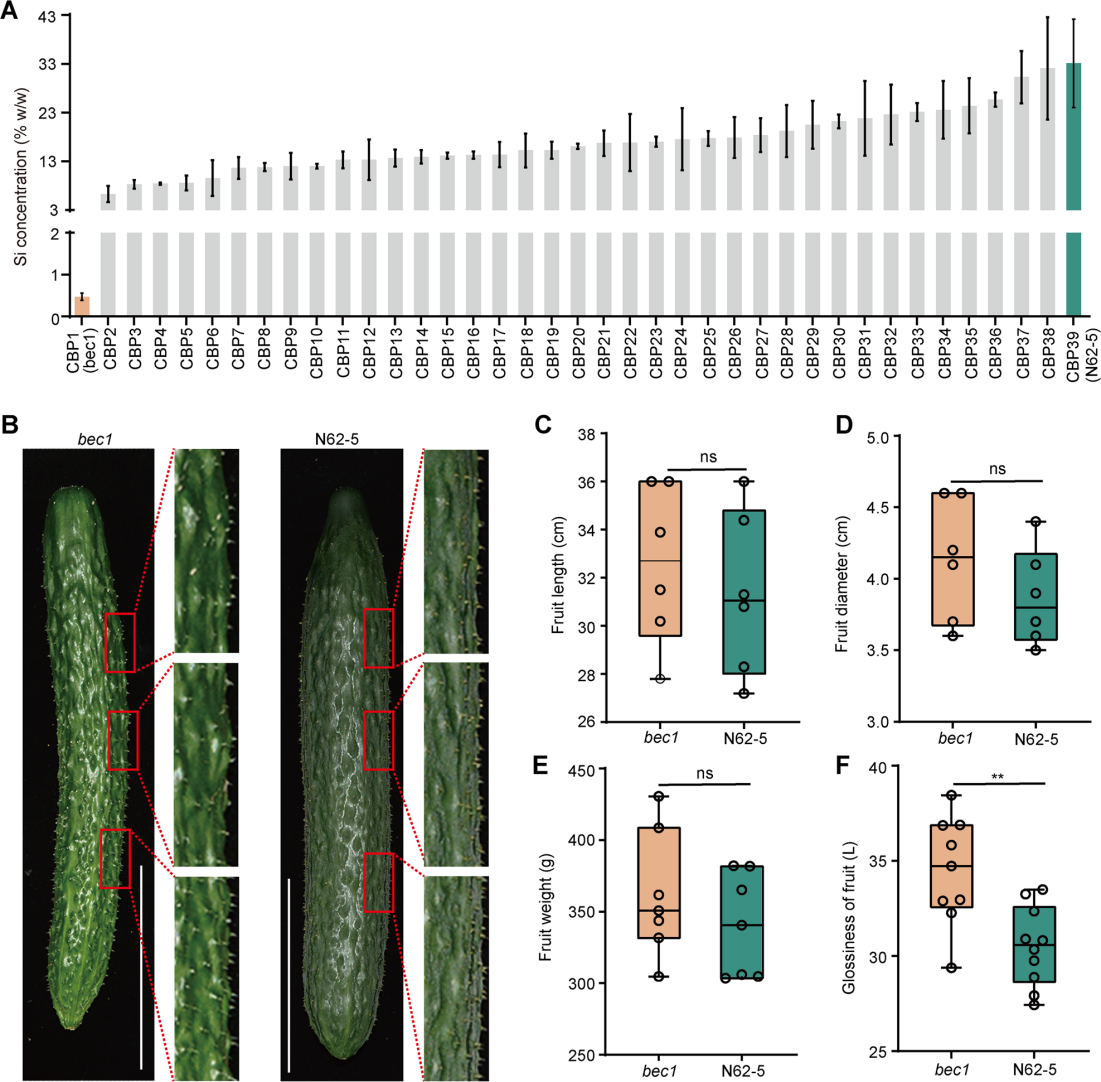
**A cucumber backbone parent line** ***bec1*** **exhibits bloomless and glossy fruit** **(A)** The mass concentration percentage of silicon (Si) in 10‐d fruits of 39 representative cucumber backbone parent lines detected by scanning electron microscopy with energy‐dispersive X‐ray spectroscopy (SEM‐EDS). The SEM‐EDS analysis was performed on the red point (spot) of the glandular trichomes, shown in Figure S1. Two‐tailed Student's *t*‐test, data are means with standard deviations (*SD*s) of three biological replicates. **(B)** The 10‐d fruit bloom phenotype of *bec1* and N62‐5. Scale bars = 10 cm. **(C–E)** The 10‐d fruit length **(C)**, diameter **(D)**, and weight **(E)** of *bec1* and N62‐5 (ns, *P* > 0.05). **(F)** Glossiness measurements of the 10‐d fruit of *bec1* and N62‐5 (**P < 0.01). For box plots, whiskers indicate the full data range, center lines indicate medians, small circles represent data from six biological replicates, two‐tailed Student's *t*‐test.

It has been reported that the glandular trichomes of cucumber release Si and polymerize it to SiO_2_ to form a layer of bloom on the surface of fruit (Samuels et al., 1991; Hao et al., 2024). To study the formation of bloom in cucumber fruit, Si deposition on the glandular trichomes of 10‐d fruits from 39 representative cucumber backbone parent lines for breeding (CBP1–CBP39) was examined using scanning electron microscopy with energy‐dispersive X‐ray spectroscopy (SEM‐EDS). The mass concentration percentage of Si in all selected lines ranged from 10% to 33%, except for line CBP1, which exhibited a negligible mass concentration percentage of Si (~0.5%) (Figures 1A, S1). To investigate the effect of Si deposits on fruit characteristics, the 10‐d fruits phenotypes of the unique CBP1 line and the line with the highest Si deposits, CBP39 (~33%, also named as N62‐5), which is a wild‐used parent line for commercial varieties, were observed (Mao et al., 2019). Phenotypic observations showed that CBP1 has bloomless fruits, while N62‐5 exhibited a prominent bloom fruits (Figures 1B, S2). Thus, the unique CBP1 line was designated as *bloomless cucumber 1* (*bec1*). Furthermore, the other fruit characteristics of *bec1* and N62‐5 were compared; the fruit length, diameter, weight, and the total wax and cutin load of *bec1* were comparable with those of N62‐5 (Figures 1C–E, S3). In contrast, colorimeter analysis showed that the glossiness value of *bec1* exceeded that of N62‐5 (Figure 1F). Thus, the cucumber backbone parent line *bec1* exhibited bloomless and glossy fruit.

### Map‐based cloning of *bec1* locus

**Figure 2:**
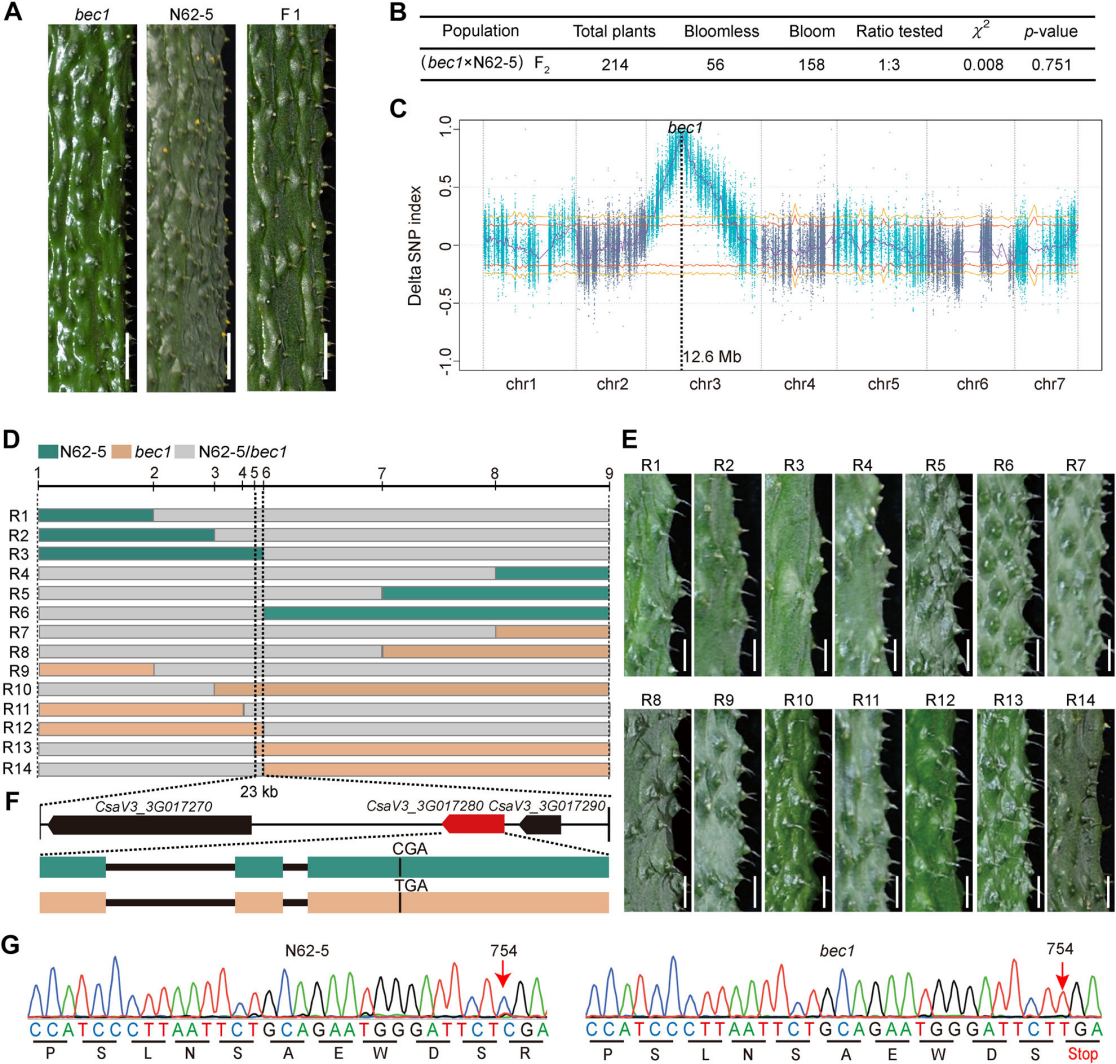
**Map‐based cloning of** ***bec1*** **locus** **(A)** The 10‐d fruit bloom phenotype of *bec1*, N62‐5 and F_1_. Scale bars = 1 cm. **(B)** Results of the *χ* ^2^ test for the segregation ratio in the F_2_ generation. The *χ* ^2^ test statistic is *χ*² = 0.008, and the *P*‐value is 0.751. **(C)** The location of the *bec1* locus revealed by bulked segregant analysis (BSA) using the ΔSNP‐index method based on the cucumber reference genome (http://cucurbitgenomics.org/ftp/genome/cucumber/Chinese_long/v1/). **(D–F)** Fine mapping of the *bec1* locus. R1–R14 represent 14 recombination types, 1–9 are SNP markers (SNP1–SNP9) **(D)**; and the 10‐d fruit bloom phenotype of R1–R14 **(E)**; the three predicted genes (*CsaV3_3G017270*, *CsaV3_3G017280*, and *CsaV3_3G017290*) identified in the 23‐kb mapping region **(F)**. Scale bars = 1 cm. **(G)** Identification of the C‐to‐T mutation at the 754th base in the coding region of *CsaV3_3G017280* in N62‐5 and *bec1* by sequencing.

To uncover the genetic locus responsible for the bloomless fruit of *bec1*, it was crossed with the bloom line N62‐5 to obtain the F_1_ cross. Phenotypic observations revealed that the 10‐d fruits of the F_1_ and N62‐5 exhibited bloom, while that of *bec1* was bloomless (Figure 2A). To further identify the genetic locus, the F_1_ was self‐crossed to generate a segregated F_2_ population. Among 214 F_2_ individuals, 56 produced bloomless fruits, while 158 exhibited bloom fruits (Figures 2B, S4). The segregation ratio of bloomless to bloom (56:158) in the F_2_ population aligned with the expected 1:3 ratio (*P* = 0.751 in the *χ* ^2^ test) (Figure 2B). Additionally, a bulked segregant analysis (BSA‐seq) was performed to pinpoint the genetic locus based on the cucumber reference genome (http://cucurbitgenomics.org/ftp/genome/cucumber/Chinese_long/v1/). The *bec1* locus is located near 12.6 Mb on chromosome 3 (Figure 2C). These results suggested that *bec1* carried a recessive genetic locus that determined bloomless fruit in cucumber.

To further determine the gene underlying the *bec1* locus, single nucleotide polymorphism (SNP) markers (SNP1–SNP9) were developed based on SNP variations in the parental sequences within this interval. Fourteen types of recombinants (R1–R14) with recombination between SNP1 and SNP9 were selected from an F_2_ population of 2,700 plants (Figure 2D). Subsequent phenotypic observations showed that the fruits of R1–R9, R11, and R14 lines were bloom; while the fruits of the R10, R12, and R13 were bloomless (Figure 2D, E). These findings suggested that the *bec1* locus could be fine mapped in the region between SNP5 and SNP6, a 23‐kb interval (Figure 2E, F).

Further sequence analysis identified three predicted genes (*CsaV3_3G017270*, *CsaV3_3G017280*, and *CsaV3_3G017290*) within the 23‐kb mapping region in the cucumber genome, which are predicted to encode mitogen‐activated protein kinase kinase kinase YODA‐like protein, citrate transporter family protein, and unknown protein, respectively (Figure 2F). Resequencing results showed that there were no sequence variations in *CsaV3_3G017270* and *CsaV3_3G017290*, including coding sequences, introns, promoters, and the 3′UTR between *bec1* and N62‐5 (Table S1). Notably, there was a C‐to‐T mutation at the 754th base in the coding region of *CsaV3_3G017280* in *bec1*, resulting in a premature termination of the encoded protein (Figure 2F; Table S1). Therefore, *CsaV3_3G017280*, here designated as *BEC1*, may be the candidate gene for the *bec1* locus.

### *BEC1* determines the formation of bloom in cucumber

**Figure 3:**
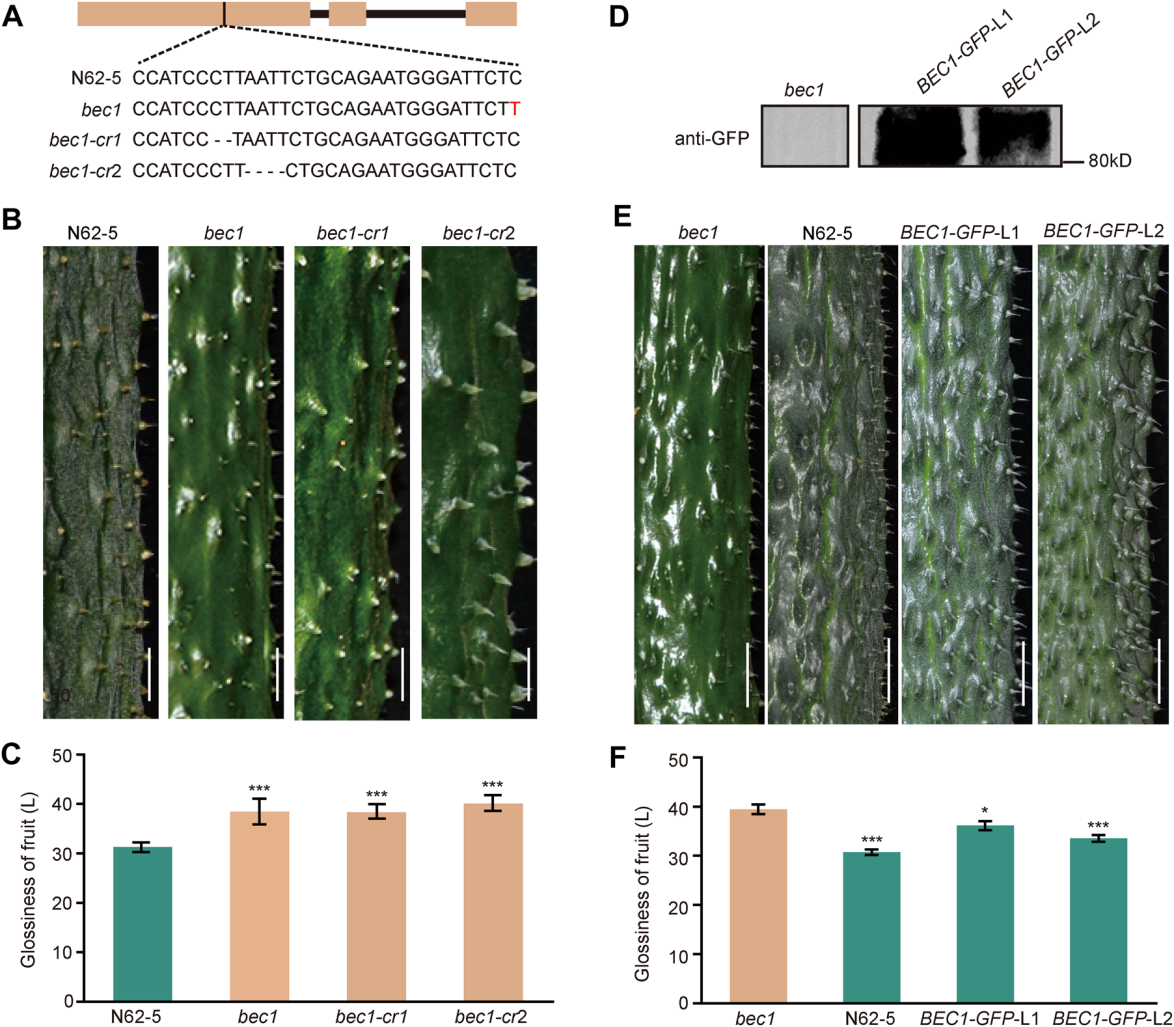
***BEC1*** **determines the formation of bloom in cucumber** **(A)** Identification of *bec1‐cr1* and *bec1‐cr2* by sequencing. **(B)** The 10‐d fruit bloom phenotype of N62‐5, *bec1*, *bec1‐cr1*, and *bec1‐cr2*. Scale bars = 0.5 cm. **(C)** Glossiness measurements of the 10‐d fruit of N62‐5, *bec1*, *bec1‐cr1*, and *bec1‐cr2* (***P < 0.001). Two‐tailed Student's *t*‐test; data are means with *SD*s of more than three biological replicates. **(D)** Identification of *BEC1::BEC1‐GFP* transgenic lines (*BEC1‐GFP*‐L1 *and BEC1‐GFP*‐L2) on the *bec1* background by immunoblotting. Total protein extracts were used for immunoblotting with anti‐GFP antibody. **(E)** The 10‐d fruit bloom phenotype of *BEC1::BEC1‐GFP* transgenic lines (*BEC1‐GFP*‐L1 *and BEC1‐GFP*‐L2) in *bec1* background and N62‐5. Scale bars = 0.5 cm. **(F)** Glossiness measurements of the 10‐d fruit of *BEC1::BEC1‐GFP* transgenic lines (*BEC1‐GFP*‐L1 *and BEC1‐GFP*‐L2) on the *bec1* background and N62‐5 (*P < 0.05, ***P < 0.001). Two‐tailed Student's *t*‐test; data are means with *SD*s of more than three biological replicates.

To determine whether *BEC1* is responsible for this locus, a target site sequence (5′‐CCATCCCTTAATTCTGCAGAATG‐3′) from the 724th to the 746th base in the coding region of *BEC1* was designed for gene editing using the CRISPR/Cas9 approach via the E‐CRISPR website (http://crispr.hzau.edu.cn/CRISPR2/) (Figure 3A). Subsequently, the loss‐of‐function mutants of *BEC1* (*bec1‐cr1* and *bec1‐cr2*) were generated to mimic the natural variation in *bec1* using the target site sequence on the N62‐5 background (Figures 3A, S5). Phenotypic observations revealed that the fruits of *bec1‐cr1* and *bec1‐cr2* were bloomless, consistent with *bec1* (Figure 3B). Additionally, colorimeter analysis showed that the glossiness values of *bec1‐cr1* and *bec1‐cr2* were comparable with *bec1*, but exceeded that of N62‐5 (Figure 3C).

Furthermore, a complementation test was performed by expressing *BEC1‐GFP* using its endogenous promoter on the *bec1* background. Two independent *BEC1::BEC1‐GFP* transgenic lines were generated, and their fruits were observed (Figure 3D). The fruits of these two lines exhibited bloom, while those of *bec1* were bloomless (Figure 3E). In addition, the fruit glossiness of these two lines and N62‐5 was lower than that of *bec1* (Figure 3F). Therefore, these results suggested that *BEC1* determined the formation of bloom to regulate the fruit glossiness in cucumber.

### *BEC1* encodes a Si efflux transporter in cucumber

**Figure 4:**
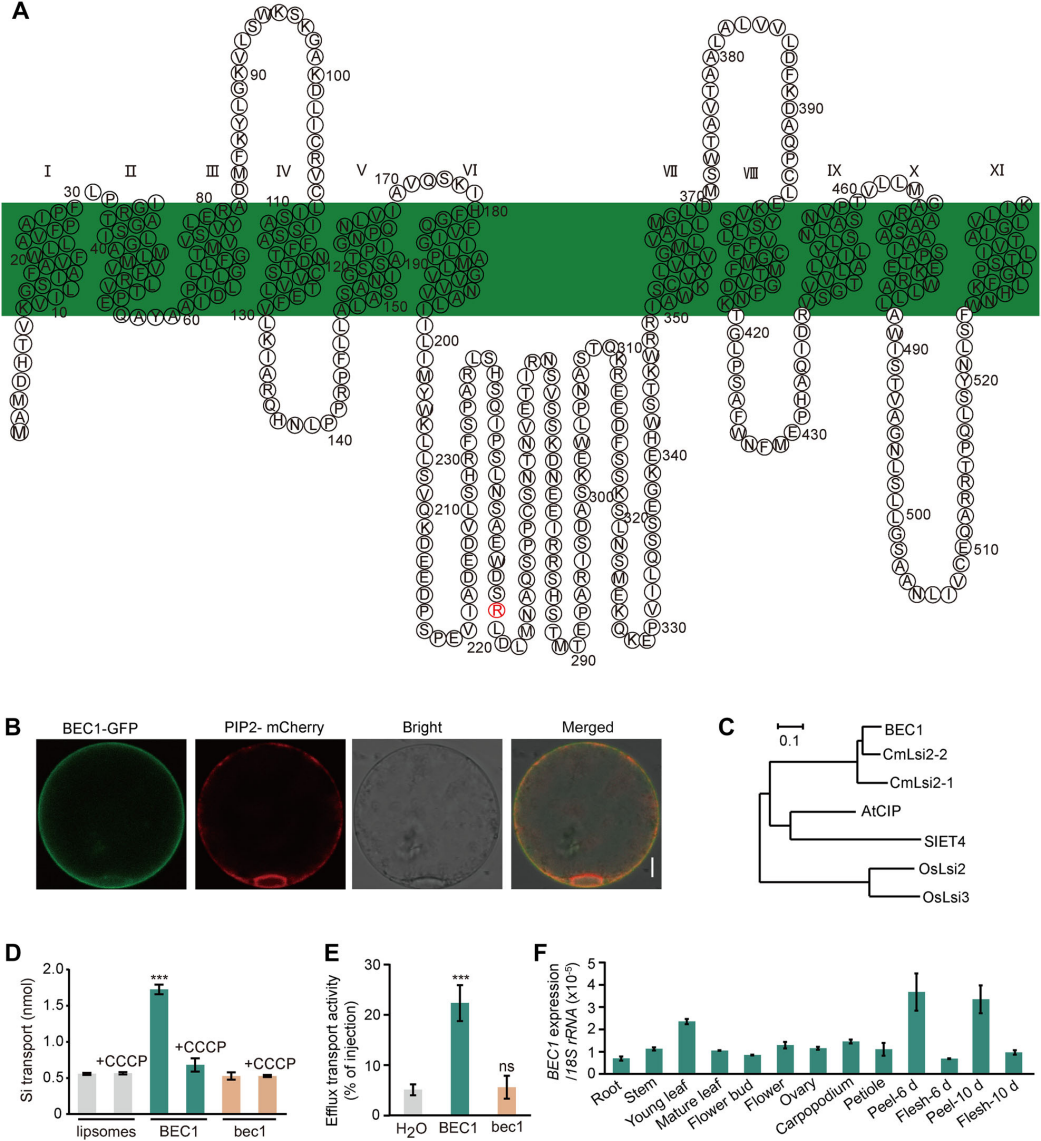
***BEC1*** **encodes a silicon (Si) efflux transporter in cucumber** **(A)** Predicted secondary structure of BEC1. Transmembrane domains have been labeled in roman numerals. **(B)** Localization of BEC1 in rice protoplasts. PIP2‐mCherry is a marker for the plasma membrane. Scale bar = 3 μm. **(C)** A phylogenetic tree of BEC1. A phylogenetic tree was constructed using the sequences of AtCIP (Arabidopsis) (Wang et al., 2024); CmLsi2‐1 (pumpkin), CmLsi2‐2 (pumpkin) (Mitani‐Ueno et al., 2011); SIET4 (rice), OsLsi2 (rice), and OsLsi3 (rice). **(D)** BEC1‐mediated Si transport activity. The proteoliposomes containing BEC1 and bec1 were incubated in a buffer solution containing 1 mM Si at pH 7.5, or pH 7.5 plus 2 μM CCCP, and assayed after 2 min. Silicon concentration was determined by ICP‐MS. Two‐tailed Student's *t*‐test; data are means with *SD*s of more than three biological replicates. **(E)** Efflux transport activity of BEC1 and bec1 in the *Xenopus* oocyte. Oocytes expressing *BEC1*, *bec1* or no expression control (water injected) were injected with 1 mM germanic acid (H_4_GeO_4_) (Ge is an analog of Si). Ge concentration was determined by inductively coupled plasma‐mass spectrometry (ICP‐MS). Two‐tailed Student's *t*‐test; data are means with *SD*s of three biological replicates. **(F)** Expression pattern of *BEC1*. The expression levels of *BEC1* in various tissues of N62‐5 was detected by quantitative real‐time polymerase chain reaction (qRT‐PCR). Two‐tailed Student's *t*‐test; data are means with *SD*s of three biological replicates.

To understand the protein function of BEC1, the protein sequence was analyzed using the SMART website (http://smart.embl-heidelberg.de/). The BEC1 is a 547‐residue protein predicted to form 11 transmembrane domains (Figure 4A). The subcellular localization assay showed that the fluorescence of BEC1‐GFP overlapped with that of PIP2‐mCherry, a marker for the plasma membrane (Figure 4B). To confirm this subcellular localization, the roots of 2‐week‐old seedlings for *BEC1*::*BEC1‐GFP*‐L1 and N62‐5 were observed using a confocal microscope. The confocal observation showed that the fluorescence of BEC1‐GFP was located in the plasma membrane, but did not exhibit polar localization (Figure S6).

To further understand the protein function of BEC1, sequence alignment with pumpkin and the model plant (Arabidopsis, rice) genome was performed, and a phylogenetic tree was constructed using the aligned protein. BEC1 was homologous to the characterized plant Si efflux transporters (Figures 4C, S7). To determine the function of BEC1, the transport activity of BEC1 for Si was tested using the proteoliposome method. BEC1 exhibited transport activity, while bec1 did not, compared with the control (liposome without protein) (Figure 4D). The efflux transport activity of BEC1 was additionally validated by expressing it in *Xenopus* oocytes. When 1 mM germanic acid (H_4_GeO_4_) (Ge is an analog of Si) was preloaded into the oocytes via micro‐injection, a significantly greater release was observed in the oocytes expressing *BEC1*. In contrast, the oocytes expressing *bec1* exhibited release levels comparable with the control group (water injections instead of cRNA) (Figure 4E).

To further determine the Si transport activity of BEC1, the roots of N62‐5, *bec1*, *bec1‐cr1*, and *bec1‐cr2* were placed in multicompartment transport boxes containing 0.5 mM silicic acid. The determination for Si exuded from the cutting end showed that the roots of N62‐5, which possess BEC1 protein, could take up and transport Si higher than *bec1*, *bec1‐cr1*, or *bec1‐cr2* (Figure S8). The expression pattern of *BEC1* was assessed using the quantitative real‐time polymerase chain reaction (qRT‐PCR) in N62‐5, and the results revealed that it was expressed in all tested tissues with higher expression levels in the peel (Figure 4F). These results suggested that *BEC1* in cucumber encoded a nonspecific expression and plasma membrane located Si efflux transporter.

### Mutation of *BEC1* inhibits the Si uptake in roots and deposition on glandular trichomes to produce bloomless fruits

**Figure 5:**
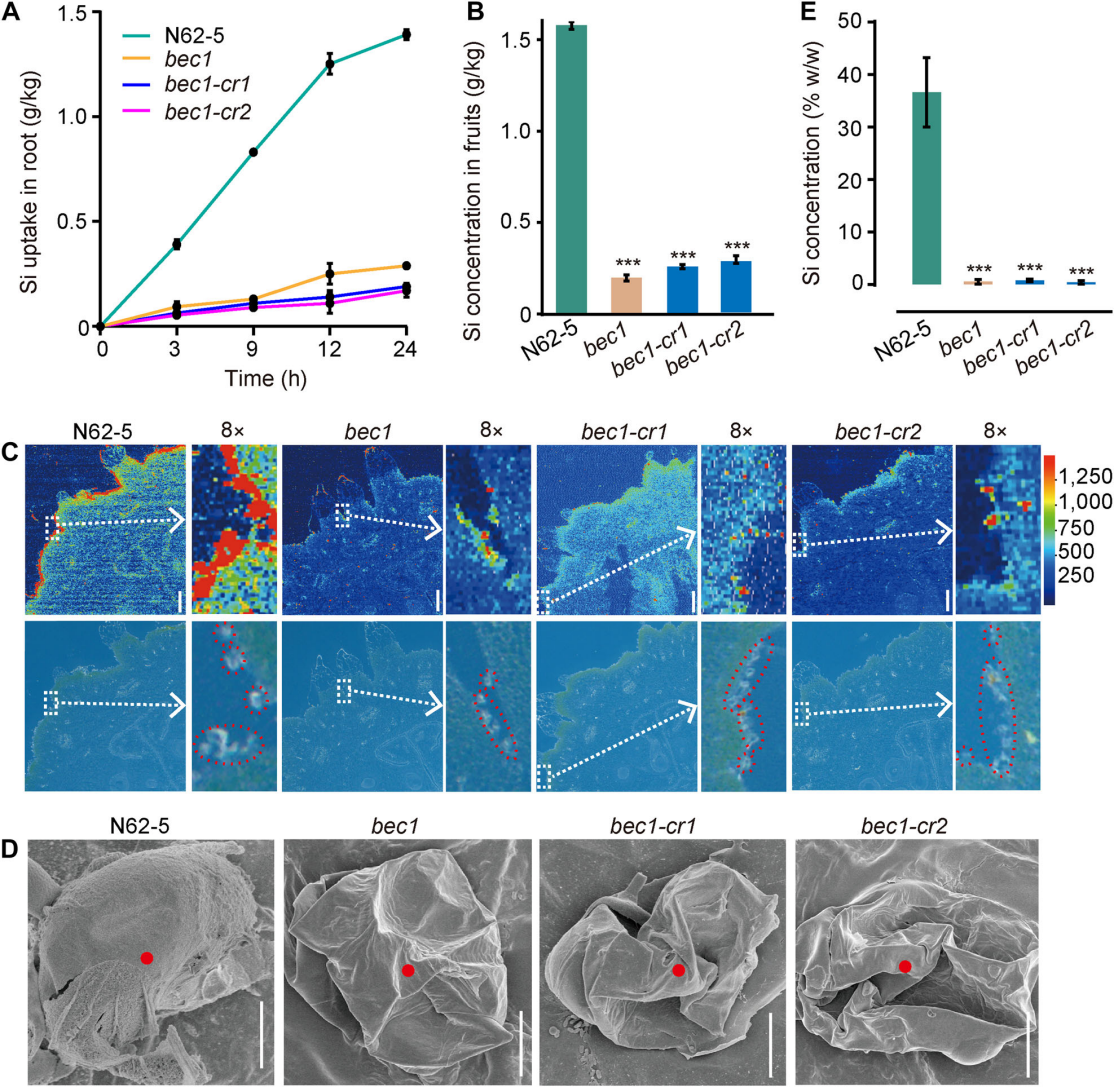
**The mutation of** ***BEC1*** **inhibits silicon (Si) uptake in root deposition on glandular trichomes to produce bloomless fruits** **(A)** Time‐dependent uptake of silicon in N62‐5, *bec1*, *bec1‐cr1*, and *bec1‐cr2* exposed to 2 mM silicic acid. Data are means with *SD*s of three biological replicates. **(B)** Silicon concentration in the fruits of N62‐5, *bec1*, *bec1‐cr1*, and *bec1‐cr2* (***P < 0.001). Two‐tailed Student's *t*‐test; data are means with *SD*s of three biological replicates. **(C)** The deposition pattern of Si in fruit of N62‐5, *bec1*, *bec1‐cr1*, and *bec1‐cr2* was detected by laser ablation inductively coupled plasma‐mass spectrometry (LA‐ICP‐MS). Scale bars = 800 μm. The white arrows show the enlarged image of the white dashed box in the figure, which has been magnified eight times. The red circles in the enlarged images highlight the glandular trichomes. **(D)** The scanning electron microscopy (SEM) images of glandular trichome on fruit of N62‐5, *bec1*, *bec1‐cr1*, and *bec1‐cr2*. Scale bars = 10 μm. **(E)** The mass concentration percentage of Si in glandular trichomes on the fruit of N62‐5, *bec1*, *bec1‐cr1*, and *bec1‐cr2* detected by SEM with energy‐dispersive X‐ray spectroscopy (SEM‐EDS). The SEM‐EDS analysis was performed on the red point (spot) of the glandular trichomes in (D). Two‐tailed Student's *t*‐test; data are means with *SD*s of three biological replicates.

To explore the effect of the BEC1 (Si efflux transporter) on bloom formation, the Si uptake capacity of the *BEC1* mutants (*bec1*, *bec1‐cr1*, and *bec1‐cr2*) exposed to 2 mM silicic acid were tested. A time‐course experiment showed that Si uptake into the roots was much lower in *bec1*, *bec1‐cr1*, and *bec1‐cr2* than in N62‐5 (Figure 5A). Subsequently, Si concentration was detected in the fruits of N62‐5, *bec1*, *bec1‐cr1*, and *bec1‐cr2*. The Si concentration of N62‐5 was 1.58 g/kg higher than that of *bec1* (0.20 g/kg), *bec1‐cr1* (0.26 g/kg), and *bec1‐cr2* (0.28 g/kg), respectively (Figure 5B). The Si deposition pattern in the fruits was further explored using a laser ablation inductively coupled plasma‐mass spectrometry (LA‐ICP‐MS) method. The majority of Si was deposited on the surface of fruit in bloom line N62‐5, and the Si deposition was impaired on the surface of *bec1*, *bec1‐cr1*, and *bec1‐cr2* (Figure 5C). Additionally, SEM‐EDS analysis of glandular trichomes revealed that the mass concentration percentage of Si was 36.6%, 0.5%, 0.8%, and 0.4% in N62‐5, *bec1*, *bec1‐cr1*, and *bec1‐cr2*, respectively (Figure 5D, E). These results suggested that mutation of *BEC1* impaired Si uptake in roots, thereby preventing deposition of Si on the surface of glandular trichomes, leading to bloomless fruits.

### The mutation of *BEC1* impairs stress resistance

**Figure 6:**
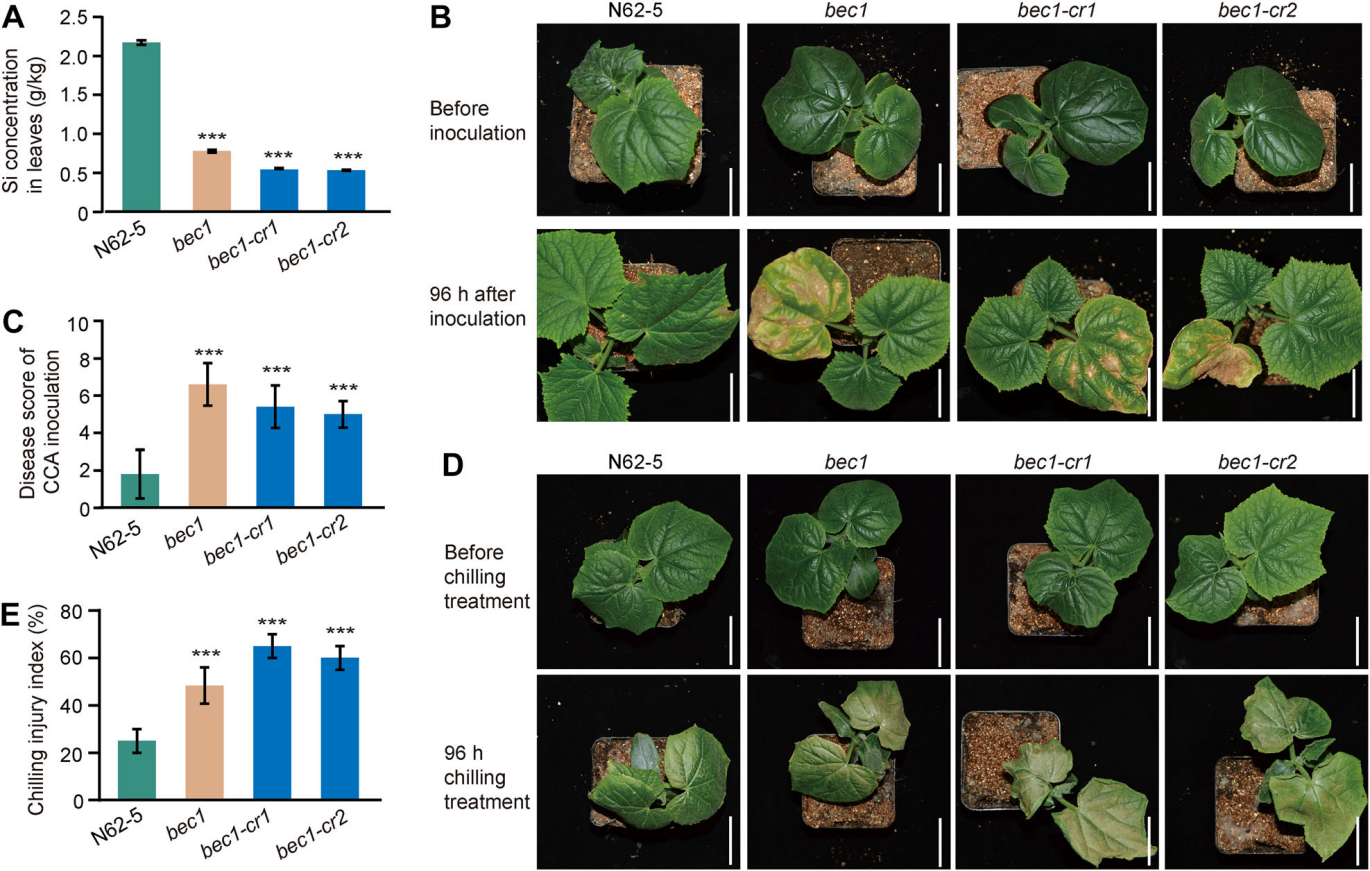
**The mutation of** ***BEC1*** **impairs the resistance to** ***Corynespora cassiicola*** **and chilling stress** **(A)** Silicon concentration in the leaves of 2‐week‐old seedlings for N62‐5, *bec1*, *bec1‐cr1*, and *bec1‐cr2* (***P < 0.001). Two‐tailed Student's *t*‐test; data are means with *SD*s of three biological replicates. **(B**, **C)** The phenotype **(B)** and disease score **(C)** of *Corynespora cassiicola* inoculation in 2‐week‐old seedlings for N62‐5, *bec1*, *bec1‐cr1*, and *bec1‐cr2*. Scale bars = 4 cm. Two‐tailed Student's *t*‐test; data are means with *SD*s of three biological replicates. **(D**, **E)** The phenotype **(D)** and chilling injury index **(E)** of 2‐week‐old seedlings for N62‐5, *bec1*, *bec1‐cr1*, and *bec1‐cr2*. Scale bars = 4 cm. Two‐tailed Student's *t*‐test; data are means with *SD*s of three biological replicates.

To test whether BEC1 affected Si accumulation in leaves, the Si concentration was measured in the leaves of 2‐week‐old seedlings of N62‐5, *bec1*, *bec1‐cr1*, and *bec1‐cr2*. The Si concentration in the leaves of N62‐5 was higher than that of *bec1*, *bec1‐cr1*, and *bec1‐cr2*, respectively (Figure 6A). Additionally, LA‐ICP‐MS was performed to assess Si deposition patterns in the leaf cross‐sections of N62‐5, *bec1‐cr1*, and *bec1‐cr2*, revealing that mutation of *BEC1* reduced Si accumulation in leaves (Figure S9). Given that plants accumulate Si for protection against stress, the deficiency in Si might impair their resistance to stress. To examine this hypothesis, the 2‐week‐old seedlings of N62‐5, *bec1*, *bec1‐cr1*, and *bec1‐cr2* were tested for responses to *Corynespora cassiicola* inoculation. Disease scoring indicated that *bec1*, *bec1‐cr1*, and *bec1‐cr2* exhibited higher susceptibility compared with N62‐5 (Figure 6B, C). Furthermore, the 2‐week‐old seedlings of N62‐5, *bec1*, *bec1‐cr1*, and *bec1‐cr2* were also tested for chilling tolerance at 4°C. The chilling injury index for *bec1*, *bec1‐cr1*, and *bec1‐cr2* exceeded that of N62‐5 (Figure 6D, E). These results suggested that mutation of *BEC1* led to deficient Si accumulation, compromising the resistance to *Corynespora cassiicola* and chilling stress.

### Grafting *bec1* scions onto rootstocks restores the deficiencies in Si and stress resistance while maintaining bloomless fruits

**Figure 7:**
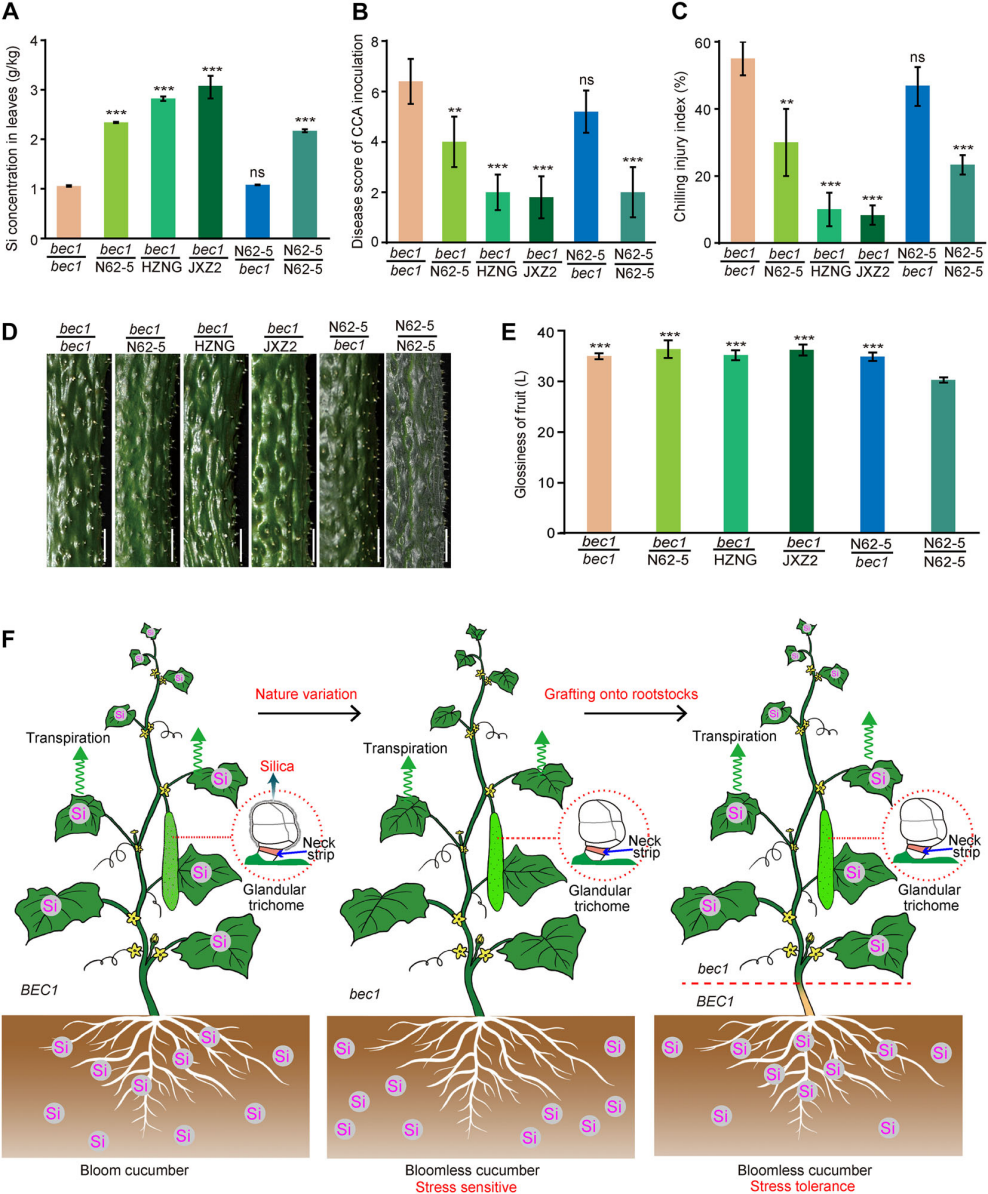
**Grafting** ***bec1*** **scions onto rootstocks restore the deficiencies in silicon (Si) and stress resistance while maintaining bloomless fruits** **(A)** Silicon concentration in the leaves of 2‐week‐old seedlings after grafting for *bec1*, *bec1* scions grafted onto rootstocks N62‐5, HZNG (*Cucurbita ficifolia* Bouché, a bloom pumpkin rootstock), JXZ2 (*Cucurbita maxima*, a bloom pumpkin rootstock), respectively, and N62‐5 and N62‐5 scion grafted onto *bec1* rootstock (***P < 0.001). Two‐tailed Student's *t*‐test; data are means with *SD*s of three biological replicates. **(B)** The disease score of *Corynespora cassiicola* inoculation in 2‐week‐old seedlings after grafting for *bec1*, *bec1* scions grafted onto rootstocks N62‐5, HZNG, BZNG, respectively, and N62‐5 and N62‐5 scion grafted onto the bec1 rootstock (**P < 0.01). Two‐tailed Student's *t*‐test; data are means with *SD*s of more biological replicates. **(C)** The chilling injury index of 2‐week‐old seedlings after grafting for *bec1*, *bec1* scions grafted onto bloom rootstocks N62‐5, HZNG, JXZ2, respectively, and N62‐5 and N62‐5 scion grafted onto the *bec1* rootstock. Two‐tailed Student's *t*‐test; data are means with *SD*s of three biological replicates. **(D)** The 10‐d fruit bloom phenotype of *bec1*, *bec1* scions grafted onto rootstocks N62‐5, HZNG, and JXZ2, respectively, and N62‐5 and N62‐5 scion grafted onto *bec1* rootstock. Scale bars = 1 cm. **(E)** Glossiness measurements of the 10‐d fruit of the scions for *bec1*, *bec1* scions grafted onto bloom rootstocks N62‐5, HZNG, JXZ2, respectively, and N62‐5 and N62‐5 scion grafted onto *bec1* rootstock (***P < 0.001). Two‐tailed Student's *t*‐test; data are means with *SD*s of more than three biological replicates. **(F)** Working model of *BEC1*. A natural SNP (C–T) variation of *BEC1* that encodes a Si efflux transporter, inhibits the Si uptake in root and deposition on the surface of glandular trichomes (GTs), resulting in bloomless fruits. At the same time, the Si deficiency impairs the resistance to stress. When the *bec1* scions were grafted onto N62‐5 or bloom pumpkin rootstocks, the Si uptake was restored, then the absorbed Si in rootstocks was transported to the leaves of scion through transpiration and rescued the resistance to stress. However, due to the presence of special “neck strips” (a type of Casparian strip) in the GTs, the loss of function of BEC1 prevents Si from crossing the “neck strips” to the surface of GTs, resulting in bloomless fruits in the scions.

To investigate potential approaches to rescue Si deficiency in bloomless cucumber, *bec1* scions were grafted onto three different rootstocks N62‐5, HZNG (*Cucurbita ficifolia* Bouché, a bloom pumpkin rootstock), and JXZ2 (*Cucurbita maxima*, another bloom pumpkin rootstock); and N62‐5 scion was grafted onto the *bec1* rootstock. The measurement of Si concentration in the leaves for 2‐week‐old seedlings after grafting showed that the Si concentration of *bec1* was less than that of *bec1* scions grafted onto each bloom rootstock, while it was comparable with that of N62‐5 scion grafted onto *bec1* rootstock (Figure 7A).

Furthermore, *Corynespora cassiicola* inoculation in grafted seedlings showed that the disease score for *bec1* was higher than that of *bec1* scions grafted onto the individual rootstock, and was comparable with that of N62‐5 scion grafted onto *bec1* rootstock (Figures 7B, S10A). Similarly, chilling treatment in grafted seedlings showed that the chilling injury index for *bec1* exceeded that of *bec1* scions grafted onto the individual rootstock, and was comparable with that of N62‐5 scion grafted onto *bec1* rootstock (Figures 7C, S10B). Notably, the fruits of *bec1* scions grafted onto these three bloom rootstocks were still bloomless, consistent with that of *bec1*, and the fruits of N62‐5 scion grafted onto *bec1* rootstock were also bloomless (Figure 7D). In addition, the fruit glossiness of these scions was higher than that of N62‐5 (Figure 7E). Collectively, these results suggested that grafting *bec1* scions onto rootstocks restored the Si deficiency and stress tolerance while maintaining bloomless fruits (Figure 7F).

## DISCUSSION

Consumable products such as fruits with a distinctly shiny appearance are enormously attractive in the market (Liang et al., 2021; Zheng et al., 2019; Yang et al., 2024; Ji et al., 2021; Mitani et al., 2011; Hao et al., 2024). Preventing the formation of bloom (means bloomless) can enhance the glossiness in plants, especially in cucumber fruit (Mitani et al., 2011; Hao et al., 2024). Here, a cucumber backbone parent line exhibited bloomless and glossy fruit, which was identified and designated as *bec1* (Figure 1). Genetic analysis showed that *bec1* carried a recessive genetic locus, which was responsible for the bloomless fruit (Figure 2A–C). The other traits of *bec1* were comparable with those of N62‐5, which is a wild‐used parent line used for commercial varieties (Figures 1C–E, S2, S3) (Mao et al., 2019). The natural SNP (C→T) variation could be developed as a molecular marker for bloomless breeding (Figure 2F, G). These results suggested that the unique *bec1* could be a critical donor parent line for bloomless breeding.

Si is deposited and polymerized to silica on the surface of glandular trichomes to form a layer of bloom in cucumber fruit (Samuels et al., 1991). In the formation of cucumber fruit bloom, an unidentified Si efflux transporter facilitates the movement of Si across the Casparian strip in root, then the Si moves upward to the shoot via the transpiration stream; finally, Si crosses the “neck strip” (a type of Casparian strip) in glandular trichomes of fruit with the help of a Si efflux transporter (Ma et al., 2007; Mandlik et al., 2020; Hao et al., 2024). Here, we identified a Si efflux transporter (BEC1) for the first time by map‐basing cloning, and was confirmed to possess Si efflux transport activity to determine the formation of fruit bloom (2, 3, 4). As BEC1 is the only Si efflux protein in the cucumber genome, it serves to facilitate Si in crossing the Casparian strip in roots and the “neck strip” in GTs simultaneously. The natural SNP (C→T) variation and gene‐editing mutation of *BEC1* led to protein dysfunction, and inhibited the movement of Si across the Casparian strip in the root and preventing Si uptake from the soil (Figures 4, 5A). Dysfunction of BEC1 also inhibited the movement of Si across the “neck strip” and prevented Si from depositing on the glandular trichomes (Figures 4, 5B–E). Without Si deposition, silica cannot be polymerized on the surface of the glandular trichomes to form bloom. Although no conserved C‐to‐T natural variation of *BEC1* in cucumber was found among homologous genes in the Cucurbitaceae using VegSNPDB (Yang et al., 2022), creating bloomless fruits can be achieved by gene editing to knock out these Si efflux proteins. Thus, the first identified BEC1 determined the formation of fruit bloom, and could be used to create bloomless fruits in crop breeding.

Si has beneficial effects on plant resistance to biotic and abiotic stresses (Mandlik et al., 2020). Previous studies have reported that Si could enhance resistance to downy mildew and *Pythium ultimum* in cucumber leaves by promoting the accumulation of phenolic compounds, or inducing phytoalexins that damage fungal hyphae (Adatia and Besford, 1986; Chérif et al., 1992; Fawe et al., 1998; Kauss et al., 2003). Si is also involved in maintaining resistance to drought, salinity and cold stresses in cucumber (Ma et al., 2004, 2023; Khoshgoftarmanesh et al., 2014; Zhu et al., 2020). However, the widely used method to produce bloomless cucumber by grafting onto bloomless pumpkin rootstocks has led to dramatic Si deficiencies, which might impair resistance to stress in cucumber scions (Seki and Hotta, 1997; Mitani et al., 2011). Our results revealed that the mutation of *BEC1* led to deficient Si accumulation, which impaired the resistance to *Corynespora cassiicola* and chilling stress (Figure 6). However, Si and stress resistance deficiency in cucumber scions could be restored by grafting onto bloom rootstocks (Figure 7A–C). The absorbed Si from rootstocks was transported to the scion leaves through transpiration (Lersten, 1997; Evert, 2006; Mandlik et al., 2020). The restored Si accumulation in the scion leaves rescued the resistance to stress. In contrast, these scions still produced bloomless fruits due to the presence of special “neck strips” in the GTs; the loss of function of BEC1 in scion prevented Si from crossing the “neck strips” to the surface of GTs (Hao et al., 2024) (Figure 7). Thus, our results implied that *BEC1* might be an ideal target for producing bloomless fruits as well as enhancing stress resistance.

In summary, a Si efflux transporter gene (*BEC1*) was isolated from a bloomless cucumber backbone parent line, *bec1*. BEC1 possessed Si efflux transport activity to facilitate the movement of Si across the Casparian strip in the root and the “neck strip” in the glandular trichomes of fruit to form fruit bloom. Mutation of *BEC1* led to Si deficiency, which impaired its resistance to *Corynespora cassiicola* and chilling stress. Interestingly, the deficiencies in Si and resistance could be restored by grafting onto rootstocks, which still produced bloomless fruits (Figure 7F). Overall, these findings are significant not only by shedding light on the mechanism of bloom formation but also in breeding, in the genetic improvement of bloomless cucumber with enhanced resistance.

## MATERIALS AND METHODS

### Genetic population and plant materials

Cucumber backbone parent lines, including N62‐5 and *bec1*, and other cucumber lines were planted in the soil of the greenhouse at the Vegetable Research Institute of the Beijing Academy of Agriculture and Forestry Sciences without Si supplements. The F_1_ generation was obtained by crossing the inbred lines N62‐5 and *bec1*, then self‐pollination to obtain the F_2_ generation.

To mutate *BEC1* using CRISPR/Cas9 technology, the target sequence (5′‐CCATCCCTTAATTCTGCAGAATG‐3′) was designed using the E‐CRISPR website (http://crispr.hzau.edu.cn/CRISPR2/). A CRISPR/Cas9 vector construction kit was used for vector construction (BGK03). The construct was transformed into the N62‐5 line. *Agrobacterium tumefaciens* strain EHA105‐mediated transformation was used to introduce the constructs into cucumber. The primer *BEC1*‐JD‐F/R (Table S2) was designed for genome PCR amplification to identify mutants.

To generate a complementation test, the genomic DNA of *BEC1*, comprising the promoter fragment and full coding sequences, was amplified and cloned into the Wmv077 vector to obtain the *BEC1::BEC1‐GFP* construct. The construct was transformed in *bec1* to generate *BEC1::BEC1‐GFP* transgenic lines.

### Scanning electron microscopy and SEM‐EDS

Silicon elemental analyses on the surface of the glandular trichomes of the 10‐d fruits (days after flowering), which were harvested from the cucumbers planted in soil of the greenhouse, were performed based on SEM observations using an S‐4800 SEM (Hitachi) with a cold field‐emission gun under an accelerating voltage of 20 kV and current of 15 mA. The SEM specimens were coated with carbon before observation. X‐ray microanalysis was performed using EDS with an ultra‐thin window detector (EX250; HORIBA) equipped with SEM.

### Resequencing and BSA‐seq analysis

The leaves of N62‐5, *bec1*, 50 bloom individual plants (mixed pool), and 50 bloomless individual plants (mixed pool) were collected for genome resequencing. DNA was extracted from the parent lines and mixed pools using DNA extraction kits. Paired‐end sequencing libraries with insert sizes of approximately 400 bp were prepared for sequencing using the extracted DNA on the Illumina NovaSeq. 6000 platform. The genome data obtained were compared with the cucumber reference genome (http://cucurbitgenomics.org/ftp/genome/cucumber/Chinese_long/v1/), and SNPs and InDels were identified from the alignment results (Li et al., 2019). The SNP‐index and differences in the offspring mixed pools were calculated. The candidate *bec1* locus was identified based on the distribution of Δ(SNP‐index) values. The Δ(SNP‐index) analysis was performed using the *QTL‐seq* R package (Magwene et al., 2011).

### Molecular marker‐assisted selection

Based on the results of BSA analysis and resequencing, SNP variations located in the target genomic region were selected to develop competitive allele‐specific PCR (KASP) markers. KASP primers were designed using flanking sequences (80 bp on either side of the SNP positions) and synthesized for the experiment. KASP markers are shown in Table S2. The PCR amplification process involved three stages: pre‐denaturation at 94°C for 15 min, denaturation at 94°C for 20 s, annealing at 61–55°C (using a touch‐down program, decreasing by 0.6°C per cycle) for 1 min, amplifying for 10 cycles, denaturation at 94°C for 20 s, annealing at 55°C, extension for 1 min, amplifying for 26 cycles.

### Transport assay

The complete coding region of *BEC1* was initially cloned into the expression vector pET‐28a. The expression and purification processes adhered to previously established protocols (Xia et al., 2021). For the transport assay, a sample of purified BEC1 (30 μg) was combined with liposomes (500 μg) and stored at −80°C for 10 min. This mixture was then diluted with a reconstitution buffer containing 20 mM MES‐KOH (pH 6.0), 0.1 M potassium acetate, and 5 mM magnesium acetate. The reconstituted proteoliposomes were collected by centrifugation at 200,000 *g* for 1 h at 4°C and then resuspended in the reconstitution buffer. The liposomes, prepared at a concentration of 10 mg/mL with a composition of 40% phosphatidylcholine, 30% phosphatidylethanolamine, 10% phosphatidylserine, and 20% cholesterol by weight, were made in a buffer containing 20 mM MES‐KOH (pH 6.0) and 1 mM DTT. For Si transport, reaction mixtures (130 μL) containing 0.5 μg of protein within the proteoliposomes, 20 mM MOPS‐KOH (pH 7.5), 0.1 M potassium acetate, 5 mM magnesium acetate, 10 mM KCl, and 1 mM Si as silicic acid were incubated at 27°C, with or without 2 μM CCCP. Based on previous reports (Mitani‐Ueno et al., 2023), the transport activity peaked at 2 min, so the assay was concluded after this time by separating the proteoliposomes from the external mixture using centrifuge columns filled with Sephadex G‐50 (fine). The Si incorporated into the liposomes was measured using ICP‐MS.

### Efflux transport activity in *Xenopus* oocytes

The efflux transport activity in *Xenopus* oocytes was performed as reported previously (Ratcliffe et al., 2017). The full‐length coding sequences of *BEC1* and *bec1* were inserted into vector pGEMHE‐mCherry with the primers listed in Table S2. The constructs were linearized with *Nhe*I, and the complementary RNA (cRNA) was synthesized with T7 *in vitro* transcription kit (mMESSAGE; Invitrogen). The isolation and culture of oocytes were performed as described previously (Ratcliffe et al., 2017). The oocytes were first injected with 30 nL/cell (800 ng/μL) of cRNA or H_2_O and cultured in ND96 solution (96 mM NaCl, 2 mM KCl, 2 mM CaCl_2_, 1 mM MgCl_2_, 5 mM HEPES, pH was adjusted to 7.5 with NaOH, supplemented with 50 μg/L ampicillin) at 18°C for 36 h. After determining protein expression using a confocal microscope, 30 nL of 1 mM germanic acid (H_4_GeO_4_) was injected into each of the expressed oocytes. The oocytes were then washed five times with ND96 and transferred to fresh ND96 at 18°C. Germanium (Ge), an analog of Si, was used as a substrate for determining Si transport activity (Mitani‐Ueno et al., 2011; Ratcliffe et al., 2017). H_4_GeO_4_ was allowed to efflux into the incubation medium. After 6 h, the incubation medium was carefully sampled and, at the end of the experiment, the oocytes were digested with concentrated HNO_3_ and the samples were analyzed for Ge by ICP‐MS.

### Silicon uptake transport activity detection

The methods for detecting Si uptake transport activity were performed as described in previous reports (Ma et al., 2001; Mitani et al., 2011). The root segments (5.5 cm long) excised from 7‐d‐old seedlings of N62‐5, *bec1*, *bec1‐cr1*, and *bec1‐cr2* were placed in a box with two compartments. The root tips (about 0–30 mm in length) were placed in the first compartment containing nutrient solution (NS10205; Coolaber, Beijing, China) and 0.5 mM silicic acid, and in the second compartment the other parts were exposed to the solution without Si. After 12 h, the Si exuded from the cutting end was determined using ICP‐MS.

### Quantitative RT‐PCR

To assess the expression levels of *BEC1* in various tissues, the root, stem, young leaf (last fully expanded leaf), mature leaf (the fifth leaf), flower bud, flower, ovary, carpopodium, petiole, peel of 6‐d fruit (days after flowering), flesh of 6‐d fruit, peel of 10‐d fruit, and flesh of 10‐d fruit were collected from the 50‐d‐old N62‐5 cucumber variety planted in soil from the greenhouse.

The total RNA of cucumber was extracted using an RNA kit (ER501; TransGen Biotech, Beijing, China). First‐strand complementary DNA was synthesized from 2 µg total RNA using a cDNA synthesis kit (R333; Vazyme, Nanjing, China). Quantitative RT‐PCR assays were performed using the SYBR Green Real‐time PCR Master Mix (QN114; Vazyme). Three biological replicates were performed for each sample. The 18S rRNA was used for the reference gene (Shimada et al., 2003). The abundance of the *BEC1* transcript in various tissues was represented as *BEC1* expression levels/18S rRNA (calculation formula: 2^−{Ct(BEC1) − Ct(18SrRNA)}^). The PCR amplification parameters were as follows: 95°C for 3 min, 95°C for 10 s, and 60°C for 30 s, totaling 40 cycles. Primers were designed using the National Center for Biotechnology Information (NCBI) and the primer sequences are shown in Table S2.

### Silicon concentration detection

To determine the Si concentration in cucumber, 0.15 g samples were weighed and mixed with 5 mL of 5% NaOH. The mixture was autoclaved at 121°C for 20 min, then filtered through a funnel. The resulting filtrate was diluted with distilled water to a final volume of 10 mL. Next, 0.5 mL of this extract was combined with 3 mL of 20% glacial acetic acid and 1 mL of 54 g/L pH 7.0 ammonium molybdate, and the reaction was allowed to proceed at room temperature for 5 min. Following this, 0.5 mL of 20% tartaric acid, 0.1 mL of 2% ascorbic acid, and 0.9 mL of glacial acetic acid were added in succession, and the reaction continued at room temperature for 30 min. Finally, the absorbance was measured at a wavelength of 650 nm.

For Si uptake capacity testing, the seeds of N62‐5, *bec1*, *bec1‐cr1*, and *bec1‐cr2* were surface sterilized, placed on wet filter paper, and germinated in an incubator at 30°C. Germinated seeds were transferred to a hydroponic system containing a Hoagland's modified nutrient solution (NS10205; Coolaber, Beijing, China), which was completely immersed in the roots and replaced every 7 d. Cucumber plants were grown in a controlled environment (28°C, 16 h d/22°C, 8 h at night and 70% relative humidity). Next, 14‐d‐old cucumber plants were treated with 2 mM silicic acid added to the nutrient solution (pH 6.0). After 0, 3, 9, 12, and 24 h of treatment, the Si content in the roots was measured.

To detect Si concentration in fruits, 10‐d fruits of N62‐5, *bec1*, *bec1‐cr1*, and *bec1‐cr2* were harvested from the cucumbers planted in the soil from the greenhouse. To detect Si concentration in leaves, the leaves were harvested from the 2‐week‐old seedlings, which were planted in a pot containing 80 g of nutritional soil without supplemental Si.

### Laser ablation ICP‐MS analysis

The LA‐ICP‐MS analysis was performed using an LA unit (GenesisGEO) equipped with a fiber femtosecond laser source operating at 343 nm using the following key settings: energy, ∼1.78% of maximum energy; scan speed, 100–500 μm/s; repetition rate, 20–50 Hz; laserspot size, 4–25 μm; and He carrier flow rate, 300 mL/min. All elemental signals were obtained with an Agilent Technologies 8900 ICP‐MS device operated in ammonia mode. Time‐course ICP‐MS output of the raster scanning was converted to an element image using iolite4 software.

### Glossiness analysis

The glossiness of the 10‐d fruits (days after flowering) was assessed using a colorimeter (HP‐200), as reported previously (Gao et al., 2023). The glossiness value, denoted as *L*, ranges from 0 to 100, representing a spectrum from black to white. Measurements of the glossiness value *L* were taken at least five times from the center of the cucumber fruit.

### Grafting method

Seedlings with one true leaf from both rootstocks and scions were prepared for grafting. First, use a blade to carefully remove the growing point of the stock and make a cut at a 45° angle approximately 0.5 cm below the cotyledon on one side of the stock. The cut reached half the diameter of the stem and was ∼1 cm long. For the scion seedlings, a diagonal cut was made about 1 cm below the cotyledon, cutting from the bottom up, with the incision depth being around two‐thirds of the stem's thickness. Cuts of the rootstocks were bound with scions tightly, then secured with a grafting clamp, and placed into a dish.

### *Corynespora cassiicola* inoculation and chilling treatment

To prepare the inoculum of *Corynespora cassiicola*, a conidia spore suspension was applied to a potato dextrose agar (PDA) plate and incubated at 25–28°C for 1–2 weeks. For inoculation, a conidia spore suspension (5 × 10^5^ spores/mL) was sprayed onto the leaves of 2‐week‐old seedlings or 2‐week‐old seedlings after grafting, which were then placed in the dark with 100% relative humidity. After 48 h, the seedlings were moved back to their normal growing environment.

For chilling treatment, the 2‐week‐old seedlings or 2‐week‐old seedlings after grafting were transferred from the regular growing incubator to the chilling incubator, which maintained a consistent temperature of 4°C.

## CONFLICTS OF INTEREST

The authors declare no competing interests.

## AUTHOR CONTRIBUTIONS

C.X., C.W., and T.W. designed the experiments and wrote the manuscript. A.M. created the backbone parent lines and genetic population. C.X., A.M., S.Y., J.Z., and R.D. performed genetic experiments. C.X., S.Y., and H.T. performed gene transformation. C.X., H.T., and Y.L. performed silicon concentration detection. C.X., C.J., and Y.L. performed transport activity detection. C.X. and C.J. performed grafting experiments and resistance to stress detection. C.W. designed all experiments, analyzed data, and wrote the manuscript. All authors discussed the results and contributed to this manuscript.

## Supporting information

Additional Supporting Information may be found online in the supporting information tab for this article: http://onlinelibrary.wiley.com/doi/10.1111/jipb.13917/suppinfo

**Figure S1.** The scanning electron microscopy (SEM) images of glandular trichome in 10‐d fruits of 39 backbone parent lines (A) and the phenotype of these fruits (B)
**Figure S2.** The 50‐d‐old plant phenotype of *bec1* and N62‐5
**Figure S3.** The wax and cutin content measurement in cucumber fruit peel of *bec1* and N62‐5
**Figure S4.** The 10‐d fruit phenotype of 214 individuals in the F_2_ population
**Figure S5.** Identification of N62‐5, *bec1‐cr1*, and *bec1‐cr2* by sequencing
**Figure S6.** Localization of BEC1 in cucumber
**Figure S7.** The sequences alignment of BEC1 with AtCIP, CmLsi2‐1, CmLsi2‐2, SIET4, OsLsi2, and OsLsi3
**Figure S8.** The Si uptake transport activity of BEC1
**Figure S9.** Deposition pattern of Si on leaf cross‐sections of N62‐5, *bec1‐cr1* and *bec1‐cr2* detected by laser ablation inductively coupled plasma‐mass spectrometry (LA‐ICP‐MS)
**Figure S10.** Grafting *bec1* onto rootstocks improves resistance to stress

**Table S1.** The variations in 23‐kb interval

**Table S2.** Primer list (5′→3′) related to experimental procedures

## Data Availability

The BSA‐seq data and the gene sequences of *BEC1* have been deposited in the Genome Sequence Archive in BIG Data Center (http://bigd.big.ac.cn), Beijing Institute of Genomics (BIG), Chinese Academy of Sciences, under the accession numbers: OMIX009304, OMIX008982, respectively.
