# Supplementary material for: The silicon efflux transporter BEC1 is essential for bloom formation and stress tolerance in cucumber
Source: J Integr Plant Biol. 2025 May 6;67(7):1895–909. doi: 10.1111/jipb.13917 (PMC12225014; doi:10.1111/jipb.13917)
Supplement: Supplementary file 1 — Figure S1. The scanning electron microscopy (SEM) images of glandular trichome in 10‐d fruits of 39 backbone parent lines (A) and the phenotype of these fruits (B) Figure S2. The 50‐d‐old plant phenotype of bec1 and N62‐5 Figure S3. The wax and cutin content measurement in cucumber fruit peel of bec1 and N62‐5 Figure S4. The 10‐d fruit phenotype of 214 individuals in the F2 population Figure S5. Identification of N62‐5, bec1‐cr1, and bec1‐cr2 by sequencing Figure S6. Localization of BEC1 in cucumber Figure S7. The sequences alignment of BEC1 with AtCIP, CmLsi2‐1, CmLsi2‐2, SIET4, OsLsi2, and OsLsi3 Figure S8. The Si uptake transport activity of BEC1 Figure S9. Deposition pattern of Si on leaf cross‐sections of N62‐5, bec1‐cr1 and bec1‐cr2 detected by laser ablation inductively coupled plasma‐mass spectrometry (LA‐ICP‐MS) Figure S10. Grafting bec1 onto rootstocks improves resistance to stress [file JIPB-67-1895-s001.docx]

**The silicon efflux transporter BEC1 is essential for bloom formation and stress tolerance in cucumber**

Changxuan Xia^1,2,3,4,6^, Aijun Mao^1,2,3,4,6^, Shanshan Yin^1,6^, Huitong Teng^1^, Caijiao Jin^1^, Jian Zhang^1,2,3,4^, Ying Li^1^, Rui Dong^1^, Tao Wu^5^ and Changlong Wen^1,2,3,4^*

1.Beijing Vegetable Research Center (BVRC), Beijing Academy of Agriculture and Forestry Sciences, Beijing 100097, China

2.State Key Laboratory of Vegetable Biobreeding, National Engineering Research Center for Vegetables, Beijing 100097, China

3.Key Laboratory of Biology and Genetic Improvement of Horticultural Crops, Key Laboratory of Superior Quality Vegetable Germplasm Innovation, Ministry of Agriculture and Rural Affairs, Beijing 100097, China

4.Beijing Key Laboratory of Vegetable Germplasms Improvement, Beijing 100097, China

5.College of Horticulture, Hunan Agricultural University, Changsha, 410128, China.

6.Co-first author

*Correspondence: Changlong Wen (wenchanglong@nercv.org).


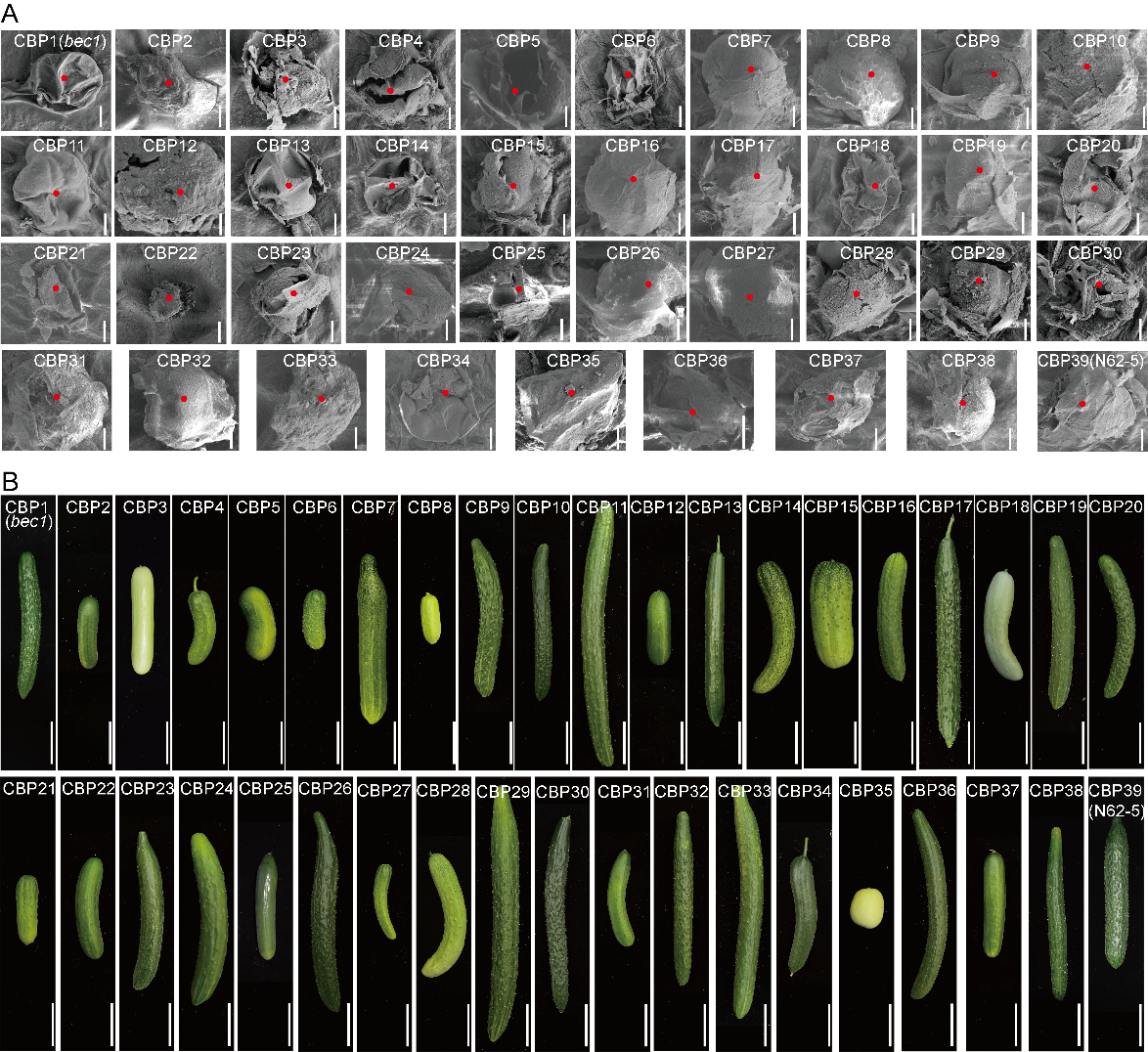


**Figure S1. The SEM images of glandular trichome in 10-day fruits of 39 backbone parent lines (A) and the phenotype of these fruits (B).** Scale bars for SEM images is 10μm, Scale bars for fruit images is 8cm.


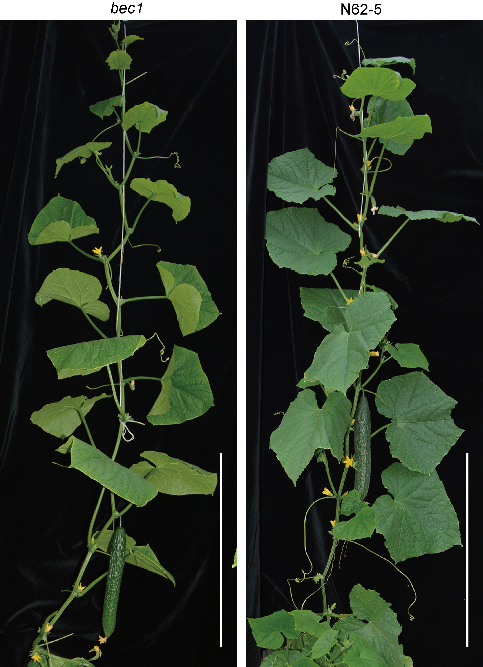


**Figure S2. The 50-day-old plants phenotype of *bec1* and N62-5.** Scale bars=50 cm.


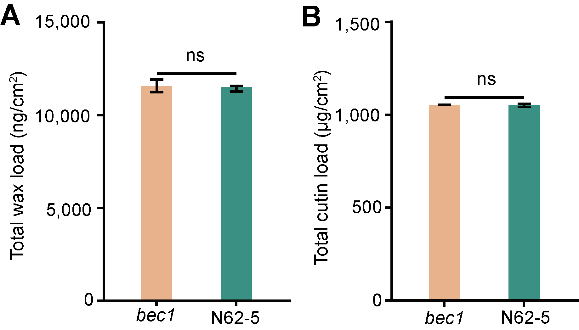


**Figure S3. The wax and cutin content measurement in cucumber fruit peel of *bec1* and N62-5.** Two-tailed Student’s *t*-test; n = 3 biological replicates


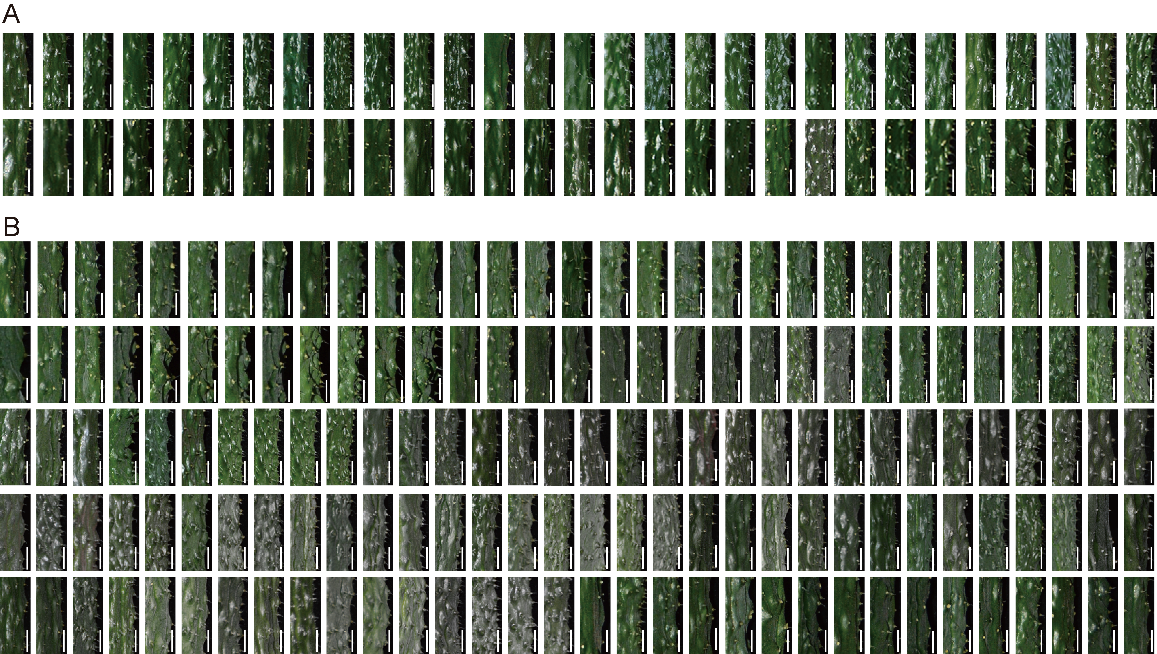


**Figure S4. The 10-day fruits phenotype of 214 individuals in the F_2_ population.** (A) The phenotype of 56 bloomless individuals. Scale bars =1cm. (B) The phenotype of 158 bloom individuals. Scale bars =1cm.


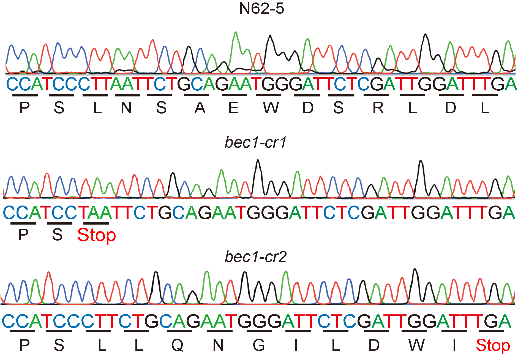


**Figure S5.** **Identification of N62-5,** ***bec1-cr1*, and *bec1-cr2* by sequencing**

**
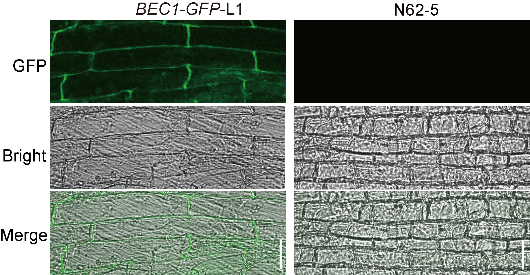
**

**Figure S6. Localization of BEC1 in cucumber.** The roots of 2-week-old seedlings for *BEC1*::*BEC1-GFP*-L1 and N62-5 were observed using confocal microscope. Scale bar=20μm.


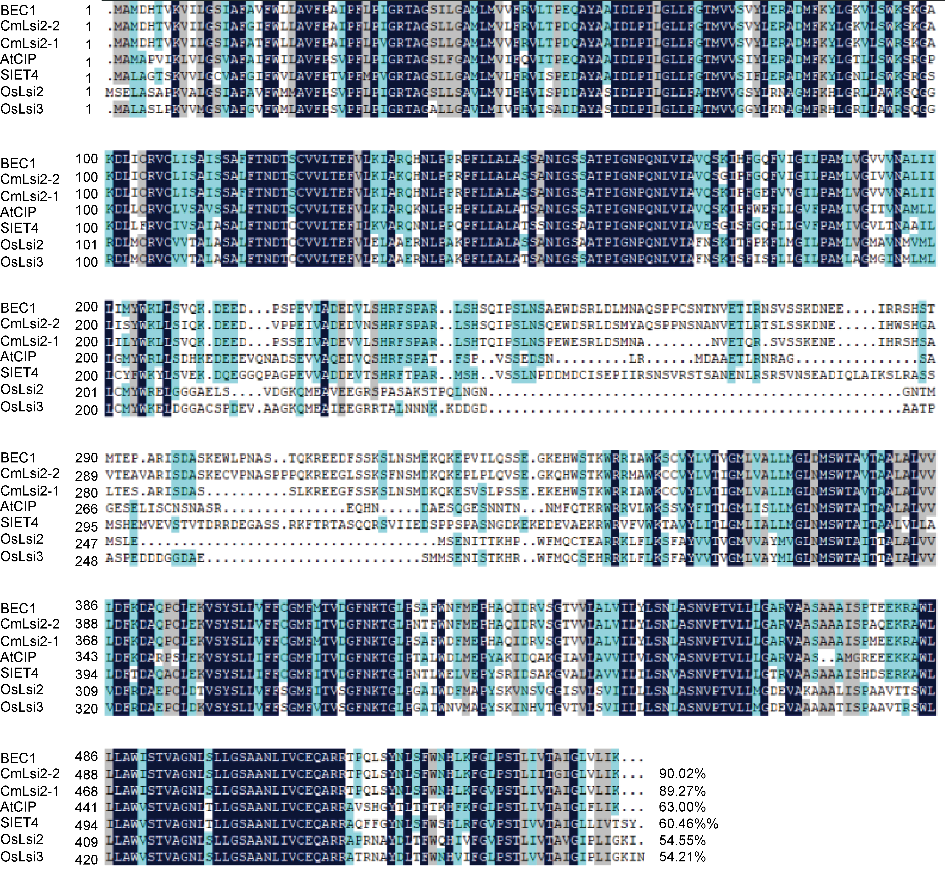


**Figure S7. The sequences alignment of BEC1 with AtCIP, CmLsi2-1, CmLsi2-2, SIET4, OsLsi2, and OsLsi3.** The percentages in the image represent the similarity of BEC1 to them.


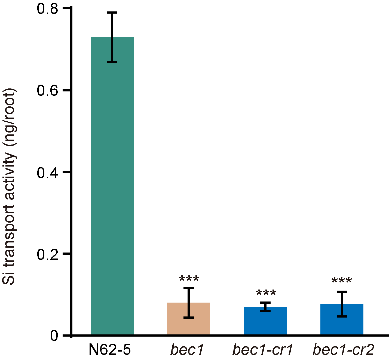


**Figure S8. The Si uptake transport activity of BEC1.** The roots of N62-5, *bec1*, *bec1-cr1* and *bec1-cr2* were placed in multicompartment transport boxes containing 0.5 mM silicic acid. The Si exuded from the cutting end was determined to assess Si uptake transport activity. Two-tailed Student’s *t*-test; Data are means with SDs of three biological replicates.


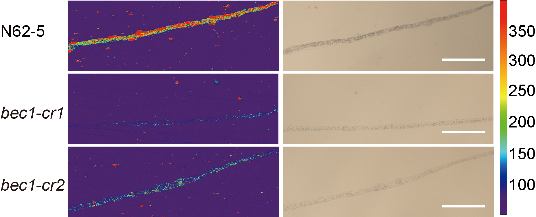


**Figure S9. Deposition pattern of Si on leaf cross-sections of N62-5, *bec1-cr1* and *bec1-cr2* detected by LA-ICP-MS**. Scale bar=2mm.


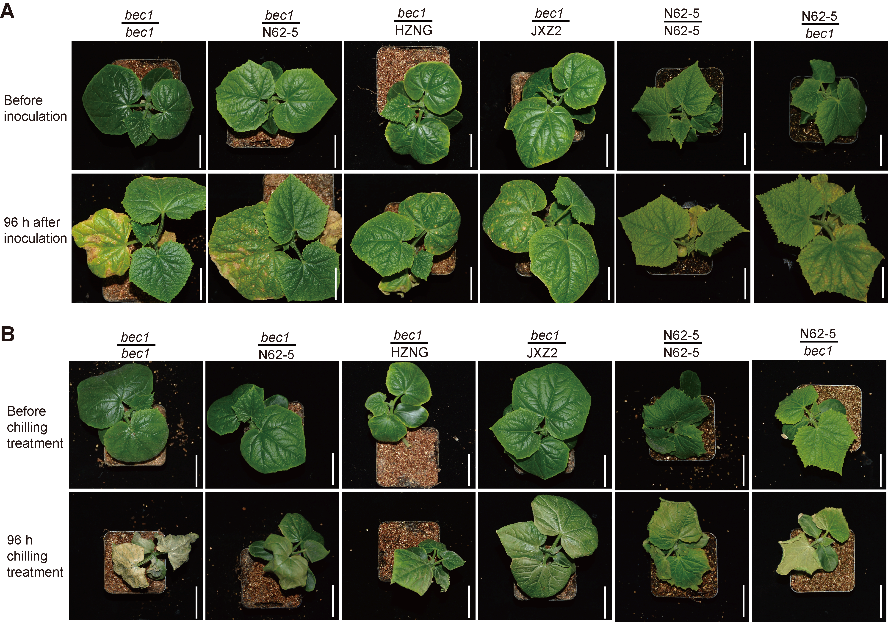


**Figure S10. Grafting *bec1* onto rootstocks improve resistance to stress.** (A and B) The phenotype of 2-week seedlings after grafting for *bec1*, *bec1* scions grafted onto bloom rootstocks N62-5, HZNG, JXZ2, respectively, and N62-5 and N62-5 scion grafted onto *bec1* rootstock; in *Corynespora cassiicola* inoculation (A) and chilling treatment (B). Scale bars=4cm.
